# A 4D-CBCT correction network based on contrastive learning for dose calculation in lung cancer

**DOI:** 10.1186/s13014-024-02411-y

**Published:** 2024-02-09

**Authors:** Nannan Cao, Ziyi Wang, Jiangyi Ding, Heng Zhang, Sai Zhang, Liugang Gao, Jiawei Sun, Kai Xie, Xinye Ni

**Affiliations:** 1 Department of Radiotherapy, The Affiliated Changzhou NO.2 People’s Hospital of Nanjing Medical University, Changzhou, 213003 China; 2 Jiangsu Province Engineering Research Center of Medical Physics, Changzhou, 213003 China; 3 Center for Medical Physics, Nanjing Medical University, Changzhou, 213003 China; 4 Key Laboratory of Medical Physics in Changzhou, Changzhou, 213003 China

**Keywords:** 4D-CBCT, Deep learning, Image quality correction, Lung cancer

## Abstract

**Objective:**

This study aimed to present a deep-learning network called contrastive learning-based cycle generative adversarial networks (CLCGAN) to mitigate streak artifacts and correct the CT value in four-dimensional cone beam computed tomography (4D-CBCT) for dose calculation in lung cancer patients.

**Methods:**

4D-CBCT and 4D computed tomography (CT) of 20 patients with locally advanced non-small cell lung cancer were used to paired train the deep-learning model. The lung tumors were located in the right upper lobe, right lower lobe, left upper lobe, and left lower lobe, or in the mediastinum. Additionally, five patients to create 4D synthetic computed tomography (sCT) for test. Using the 4D-CT as the ground truth, the quality of the 4D-sCT images was evaluated by quantitative and qualitative assessment methods. The correction of CT values was evaluated holistically and locally. To further validate the accuracy of the dose calculations, we compared the dose distributions and calculations of 4D-CBCT and 4D-sCT with those of 4D-CT.

**Results:**

The structural similarity index measure (SSIM) and peak signal-to-noise ratio (PSNR) of the 4D-sCT increased from 87% and 22.31 dB to 98% and 29.15 dB, respectively. Compared with cycle consistent generative adversarial networks, CLCGAN enhanced SSIM and PSNR by 1.1% (p < 0.01) and 0.42% (p < 0.01). Furthermore, CLCGAN significantly decreased the absolute mean differences of CT value in lungs, bones, and soft tissues. The dose calculation results revealed a significant improvement in 4D-sCT compared to 4D-CBCT. CLCGAN was the most accurate in dose calculations for left lung (V5Gy), right lung (V5Gy), right lung (V20Gy), PTV (D98%), and spinal cord (D2%), with the relative dose difference were reduced by 6.84%, 3.84%, 1.46%, 0.86%, 3.32% compared to 4D-CBCT.

**Conclusions:**

Based on the satisfactory results obtained in terms of image quality, CT value measurement, it can be concluded that CLCGAN-based corrected 4D-CBCT can be utilized for dose calculation in lung cancer.

## Introduction

Radiation therapy is one of the important methods of treating cancer. However, radiation may cause side effects on surrounding normal tissues, especially in organ treatment with precise positioning of respiratory motion, such as liver, lung, and mediastinum [1, 2]. In addition to intensity-modulated radiotherapy for lung cancer, stereotactic body radiotherapy (SBRT) is clinically applied for early-stage non-small cell lung cancer patients who are unsuitable for or refuses surgery [3, 4]. SBRT requires a large single dose, is more challenging in positioning and treatment. Using only three-dimensional (3D) imaging can cause the blurring of anatomical structures, whereas four-dimensional (4D) imaging can dynamically display the movement of organs in radiotherapy [5]. When the target position is affected by respiratory motion, the utilization of 4D-CT for localization and treatment planning can minimize the impact of respiratory-induced uncertainties on the displacement of the target position. Subsequently, the choice of a 4D-CBCT has practical significance for the repetition of target area location and dose during treatment [6]. Meanwhile, adaptive radiotherapy (ART) based on CBCT, which changes the treatment plan according to the transformation of the target area during sub-treatment, has clinical significance [7, 8]. However, the relevant studies are primarily limited to 3D-CBCT at present. Studies have shown that 4D-CBCT and 4D-CT used for adaptive radiotherapy (ART) can mitigate the impact of interfractional changes while reducing the PTV volume and minimizing radiation dose to normal tissue [9–11]. Harsolia et al. [9] compared various planning techniques including 3D-conformal, 4D-union, 4D-offline adaptive, and 4D-online adaptive to enhance the accuracy and decrease the planning target volume (PTV) margin in image-guided radiotherapy using 4D-CBCT. The results revealed that 4D-CBCT is more effective in guiding adaptive radiotherapy than 3D-CBCT. Nonetheless, 4D-CBCT suffers from low image contrast and poor quality due to the undersampling of the projections of each temporal phase [12]. Additionally, issues such as scatter artifacts, image lag, beam hardening, and patient movement during acquisition result in distorted CT values [13]. ART is a promising vision for the future, and these challenges present hurdles to the clinical implementation of 4D-CBCT for dose calculation if it were to be used in ART [9, 11].

Research in the field of CBCT value correction is primarily based on three types of artifacts: scatter, motion, and streak artifacts. The correction of scatter artifacts can be achieved through Monte Carlo simulation [14], which involves simulating the transmission, scattering, and absorption of X-rays in human tissues to improve the accuracy of CBCT dose calculation. However, the motion artifacts remaining in CBCT can cause blurring of tumors and tissues within the lungs. In addition to correcting motion artifacts, the use of 4D-CBCT in clinical practice effectively reduces the generation of motion artifacts but inevitably causes streak artifacts due to undersampling. To reduce streak artifacts, Li [15] et al. improved the image quality by increasing the scanning time and scanning dose, but it results in increased patient irradiated dose and reduced clinical efficiency. Accordingly, some studies use iterative algorithms such as total variation regularization [16] and non-local means [17] to protect the edges of the image and suppress noise. Wang et al. [18] proposed motion-compensated reconstruction based on prior knowledge to improve image quality. Considering the repetitiveness of patient respiratory motion, Huang et al. [19] optimized the registered deformation vector field (DVF) on this basis to further improve the efficiency and accuracy of reconstruction. In recent years, deep learning has been extensively used in medical-image classification, segmentation, denoising, and super-resolution reconstruction. It is also gradually being used in the image correction of 4D-CBCT. The primary application approaches include deep-learning models combined with other correction methods (4D-AirNet (2020) and CNN-MoCo (2023)) and deep-learning network models only. Given the over-smooth of image edges and contrast reduction caused by iterative algorithms, Jiang et al. [20] proposed SR-CNN (2018) to improve the sharpness of edges and anatomical structure details in undersampled CBCT. Sun [21] et al. proposed a model of U-net combined with transfer-learning strategy (2020). It uses transfer learning to fine-tune the 4D-CBCT enhanced by U-net, resulting in significant improvements in structural similarity index measure (SSIM) and peak signal-to-noise ratio (PSNR) compared with before fine tuning. Later, the RDN residual dense network (2020) proposed by Madesta [22] et al. simulates streak artifacts to achieve correction of 4D-CBCT without affecting the anatomical information.

The correction of 4D-CBCT by generating 4D-sCT is a research hotspot. Thummerer et al. [23] used deep convolutional networks to generate synthetic CT (sCT) through paired training of a single-phase image for dose calculation in lung-cancer radiotherapy. Considering that the training depends on the reproducibility of patient's breathing, 4D-CBCT cannot use paired supervised data for model training. Usui et al. [24] used cycle consistent generative adversarial networks (CycleGAN) for the unpaired training of images from two thresholds, 4D-CT and 4D-CBCT. However, due to the limited training data and training with only a single time phase during training, some bones are not fully recovered. The robustness also requires further improvement.

In the present study, 4D-CT and 4D-CBCT were paired trained in a network called contrastive learning (CL)-based cycle generative adversarial networks (CLCGAN), which combined the latest CL and CycleGAN [25–27]. CLCGAN was used to explore the mutual information present in 4D-CT and 4D-CBCT during training, aiming to train a model capable of generating images with reduced streak artifacts. Ideally, CLCGAN selectively generates images with high similarity in the feature space. To evaluate the model performance, quality and CT values were quantitatively assessed, and the accuracy of dose distribution and calculation of generated images was verified.

## Materials and methods

### Patient data

4D images of 20 patients with thoracic tumors were selected to train and test the deep-learning model. Patient data were obtained from a publicly available dataset in the Cancer Imaging Archive (TCIA, http://www.cancerimagingarchive.net/) created by the National Cancer Institute [28, 29]. All the patients had locally advanced non-small cell lung cancer and received concurrent chemoradiotherapy, with a total dose ranging from 59.4 to 70.2 Gy delivered in daily 1.8 or 2 Gy fractions. All patient clinical information used for training and testing is shown in Table 1. Throughout their treatment, the patients all underwent 4D-CT imaging at least once and most received 4D-CBCT imaging during treatment fractions. Consequently, the dataset consisted of a total of 82 4D-CT and 507 4D-CBCT images from these 20 patients.

**Table 1:** Clinical information for 20 patients

Patient	Overall stage	Location	Tumor volume(cc)	Patient	Overall stage	Location	Tumor volume(cc)
1	IIIA	LUL	18	11	IIIA	RUL	13
2	IIIA	RUL	12	12	IIIB	RUL	142
3	IIIB	RLL	392	13	IIIB	RUL	75
4	IIIB	RLL	55	14	IIIB	RUL	27
5	IIIA	RLL	75	15	IIIB	LUL	171
6	IIIA	RUL	31	16	IIIB	RUL	58
7	IIIA	RLL	179	17	IIIB	LLL	47
8	IIIB	RUL	7	18	IIIA	LLL	33
9	IIIA	LUL	33	19	IIIA	mediastinum	143
10	IIIA	RUL	10	20	IIA	LLL	78

The table lists the clinical overall stage, tumor location and tumor volume of 20 patients. And the tumor locations including right upper lobe(RUL), right lower lobe(RLL), left upper lobe(LUL), left lower lobe(LLL) of lung and mediastinum

### Image data

#### 4D-CT

4D-CT images were acquired on a 16-slice helical CT simulator (Brilliance Big Bore, Philips Medical Systems, Andover, MA, USA) under scanning conditions with a tube voltage of 120 kVp, tube currents of 50–114 mA, and exposure times of 3.53–5.83 ms. The respiratory signals obtained from the RPM respiratory gating system were divided into 10 phases from 0 to 90% in phase order, with the 0% phase corresponding to the end of inspiration. The slice thickness for each phase was 3 mm, and the image size was 512 × 512 with a pixel spacing of 0.9766 × 0.9766 mm^2^.

#### 4D-CBCT

4D-CBCT images were acquired on a commercial CBCT scanner (On-Board Imager v1.3, Varian Medical Systems, Inc.) with 360° scanning at a tube voltage of 125 kVp, a tube current of 20 mA, and an exposure time of 20 ms. To promise the appropriate calculation of radiotherapy dose, CT number to electron density (CT-ED) calibration was performed with a CIRS (Norfolk, Virginia, US) phantom named Model 062M Electron Density Phantom on 4D-CBCT. During scanning, the respiratory surrogate used for 4D-CT were integrated into the 4D-CBCT acquisition system. The projection was sorted into the same 0–90% phases according to respiratory signal of surrogate. Each phase was reconstructed using the Feldkamp–Davis–Kress reconstruction algorithm with a slice thickness of 3 mm, an image size of 512 × 512, and a pixel spacing of 0.8789 × 0.8789 mm^2^.

### 4D-sCT based on CLCGAN

#### Image preprocessing

The training dataset comprised 4D images of 10 phases from 20 patients. Each phase comprised 50 slices, with a total of 10,000 4D-CT and 10,000 4D-CBCT slices. Each patient was centered on the lung cancer region, including the whole lung. Each phase of 4D-CT images were adjusted to the same size and resolution as the 4D-CBCT images using an open-source registration tooltik, elastix [30, 31]. The adjusted images were used for paired training with CLCGAN, and random flipping was applied during training to achieve data augmentation.

#### Network architecture

**Fig. 1:**
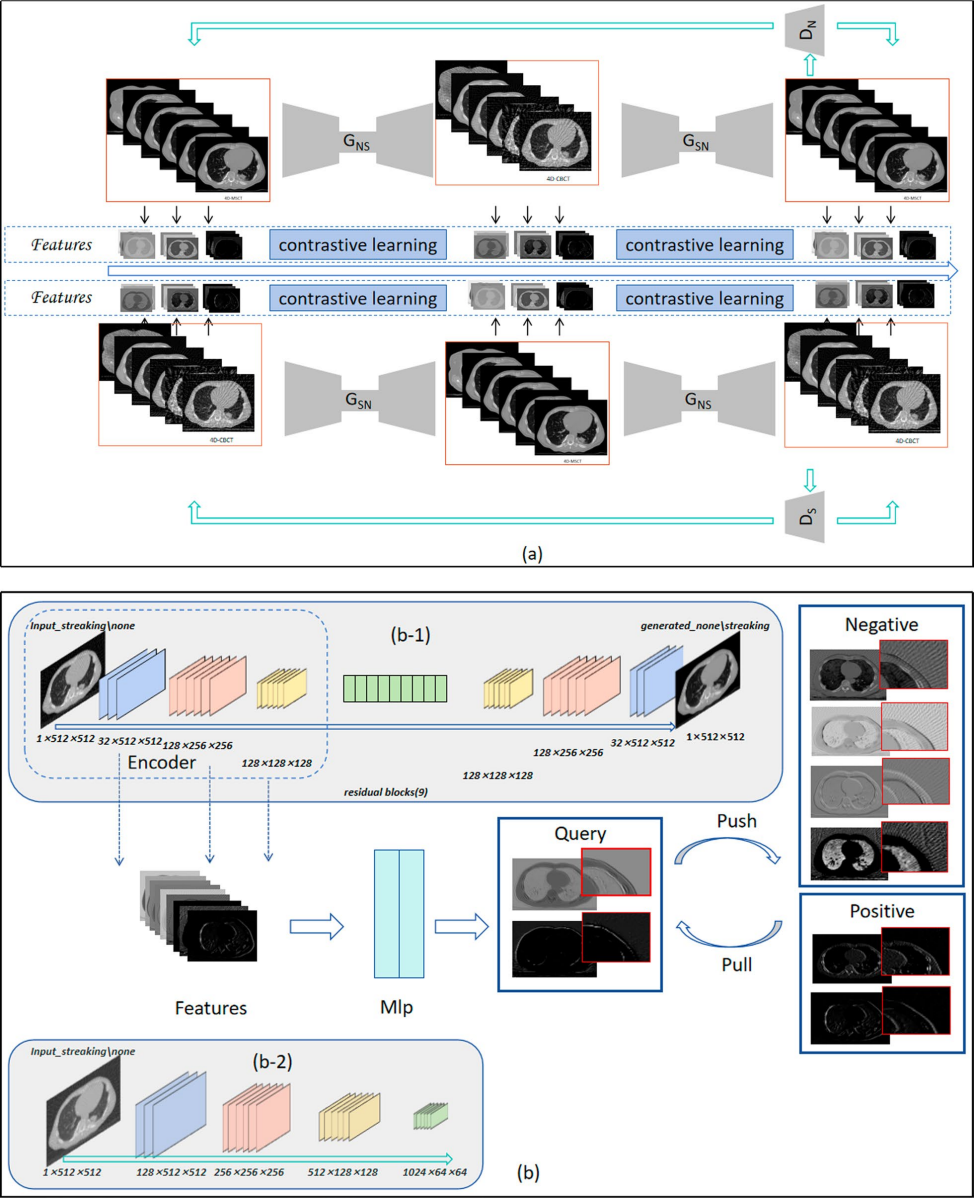
Architecture and module details of the CLCGAN network: **a** illustrates the overall architecture of CLCGAN; and **b** showcases the detailed principle of contrastive learning, where (b−1) and (b−2) show the internal diagrams of the generator and discriminator, respectively

The CLCGAN network model applied the idea of CL to the dual-domain CycleGAN. It used only the similar features in the dual domain for image generation to realize the removal of streak artifacts. Therefore, CLCGAN comprised two branches: CycleGAN and CL. CycleGAN realized the mutual mapping of CBCT/CT to CT/CBCT to obtain the feature information of two samples. CL implemented constraints on the feature space to better guide image generation. Figure 1a shows the network architecture of CLCGAN. The implementation details of these two branches are described as follows.

**Fig. 2:**
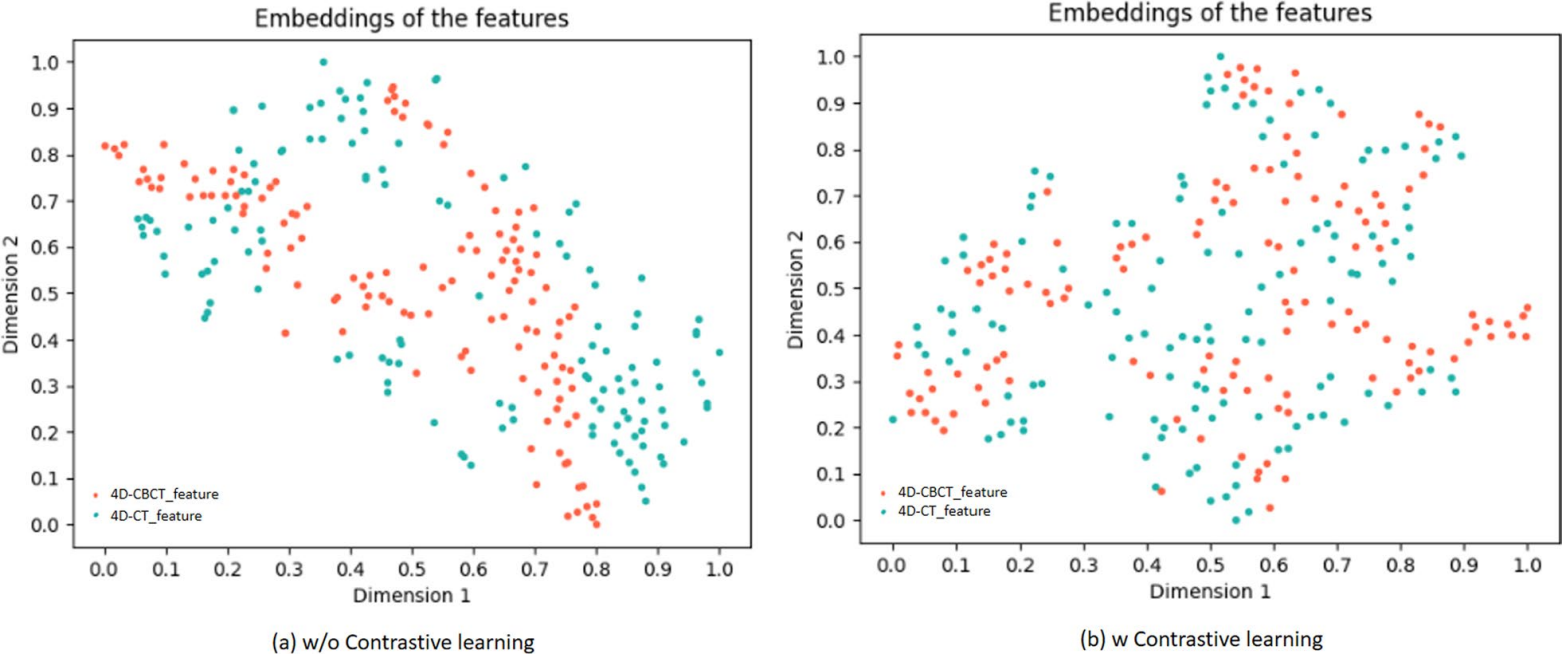
t-SNE Plots of Learned Features with and without CL. **a** and **b** represent the feature distribution obtained without and with the incorporation of contrastive learning, respectively

CycleGAN contained two symmetric sub-networks for generating 4D-sCT (CT → sCBCT → sCT) and 4D-sCBCT (CBCT → sCT → sCBCT). Each sub-network comprised two generators and one discriminator. Figure 1b shows the architecture of generators, where each generator comprised a three-layer encoder, a nine-residue block structure, and a three-layer decoder, whereas the discriminator comprised a four-layer encoder. The two sub-networks were simultaneously trained to extract features from CBCT and CT and thus form a feature space for regularization. The network performance was improved by optimizing the loss function between the generated and original image until the discriminator cannot distinguish between sCT, sCBCT and CT, CBCT, the model tends to converge. Ultimately, the removal of streak artifacts in 4D-CBCT was achieved by generating 4D-sCT, although the effect of artifact removal was weak. Accordingly, we combined CL to constrain the feature space and realize streak artifacts removal in latent space. CL is an unsupervised learning. The main idea is to set low-difference features with similar or common properties in CBCT and CT to “positive” and vice versa to “negative”. During training, only “positive” features were used for image reconstruction or image recovery. To maintain the model architecture, features were directly extracted from the encoder of the generator, and the features from each layer were sent to a two-layer multilayer perceptron. In the feature embedding space, the feature $\hat{x}$ from one side of the CT or CBCT served as a query, whereas the other side contained the positive feature $\hat{x}^{ + }$ and k negative feature {$\hat{x}_{i}^{ - }$}$_{{{\text{i}} - 1}}^{{\text{k}}}$. Positive features were proximity to query, so they were correlated with each other (none streaking →  ← none streaking); otherwise, they were detached from each other (streaking ←  → none streaking). To visualize the impact of CL, the features extracted for image generation with and without CL were visualized using t-distributed Stochastic Neighbor Embedding (t-SNE) [32]. Results are shown in Fig. 2. The two features had closer distances and overlapped more after using CL. When using t-SNE to compare two features, if there is some degree of similarity between the two features, the corresponding data points in the t-SNE's two-dimensional coordinates will completely overlap and embed each other, rather than exhibiting distinct boundaries. Therefore, the features selected for generating the sCT were free of streak artifacts.

#### Loss function

In the experiment, the final loss function included a loss function $L_{cont}$ for enforcing the distribution of the specified features, a loss function $L_{adv}$ for minimizing the difference between the expected and predicted values of 4D-CT/4D-CBCT, and a loss function $L_{cyc}$ for minimizing the difference between the original images of 4D-CT/4D-CBCT and the generated images. To further preserve the structure and content information of the images, a frequency loss function $L_{freq}$ was utilized to fully leverage the frequency domain information. The overall loss function is represented as

1 $$L_{{{\text{total}}}} = \lambda_{1} L_{cont} + \lambda_{2} L_{cyc} + \lambda_{3} L_{adv} + \lambda_{4} L_{freq} ,$$

$\lambda_{i}$ is the weight parameter for each item, and we set $\lambda_{i}$_,_ $\lambda_{2}$_,_ $\lambda_{3}$ and $\lambda_{4}$ to 2, 1, 1, and 0.01 respectively.

Contrastive loss: The feature was normalized to $f = E(\hat{x})$_,_ $f^{ + } = E(\hat{x}^{ + } )$_,_ $f_{{\text{i}}}^{ - } = E(\hat{x}_{i}^{ - } )$ by formula, and the function of the canonical feature distribution is denoted as

2 $$\begin{gathered} L_{{{\text{cont}}}} {\text{(G}}_{SN}, {\text{G}}_{NS} ) = E_{s\sim S,n\sim N} \left[ { - {\text{log}}\frac{{{\text{sim}}(f,f^{ + } )}}{{{\text{sim(}}f,f^{ + } ) + \sum\nolimits_{{{\text{i}} = 1}}^{{\text{N}}} {{\text{sim(}}f,f_{i}^{ - } {)}} }}} \right], \hfill \\ \hfill \\ \end{gathered}$$

3 $${\text{sim}}({\text{u}},{\text{v}}) = {\text{exp}}\left( {\frac{{u^{\tau } v}}{{\left\| {\text{u}} \right\|\left\| {\text{v}} \right\|\tau }}} \right),$$

sim(u, v) represents the cosine similarity function between two normalized feature vectors, and τ represents the temperature parameter, which is set to 0.07.

Adversarial loss: The discriminator $D_{N}$/$D_{S}$ was trained to make the discriminating output on 4D-CT/4D-CBCT close to 1 and the generated 4D-sCT/4D-sCBCT image output close to 0. Minimize $L_{adv}$. Thus, the final generator made the output of discriminator on generated images as close to 1 as possible. Therefore, the adversarial loss function is denoted as

4 $$L_{adv} (G_{SN}, {\text{D}}_{N} ) = E_{n\sim N} \left[ {\log D_{N} ({\text{n}})} \right] + E_{s\sim S} \left[ {\log (1 - D_{N} (G_{SN} ({\text{s}})))} \right],$$

Cycle consistency loss: The generator $G_{SN}$/$G_{NS}$ was trained to minimize $L_{cyc}$ so that the difference between the generated image and the real sample s/n was minimized. The cycle consistency loss function is denoted as

5 $$L_{{{\text{cyc}}}} = E_{n\sim N}^{{}} \left[ {\left\| {G_{SN} \left( {G_{NS} \left( n \right)} \right) - n} \right\|_{1} } \right] + E_{s\sim S}^{{}} \left[ {\left\| {G_{NS} \left( {G_{SN} \left( {\text{s}} \right)} \right) - s} \right\|_{1} } \right],$$

Frequency loss:

6 $$L_{freq} = E_{n\sim N} [||FT(G_{S2N} (G_{N2S} (n))) - FT(n)||_{2}^{2} ] + E_{r\sim R} [||FT(G_{N2S} (G_{S2N} (r))) - FT(r)||_{2}^{2} ],$$

#### Parameter selection

During training, a batch size of 1 and instance normalization were used. The training images were randomly cropped into 512 × 512 blocks in a paired manner for CL. In the training process, Adam optimizer with parameters $\beta_{1}$ = 0.5 and $\beta_{2}$ = 0.999 and a learning rate of 0.0002 were adopted, and the model was trained for 100 epochs starting from 0. The entire network based on the PyTorch framework was implemented on a deep-learning server (Inter (R) Xeon (R) Gold 6133 CPU @ 2.50 GHz, NVIDIA A100 80 GB, 256 GB).

### Evaluation methods

#### Image-quality assessment

To evaluate the effect of the CLCGAN model in removing image artifacts, we selected five cases comprising 2500 untrained paired 4D-CT and 4D-CBCT slices for testing. The resolution and size of the testing data were kept consistent with the training data. The evaluation comprised two parts: comparing the generated 4D-sCT with the original 4D-CT, and comparing the 4D-sCT generated using the CLCGAN and CycleGAN network individually.

To quantitatively evaluate the image quality, the 4D-CBCT, 4D-sCT based on CycleGAN, and CLCGAN were measured against the original 4D-CT by using SSIM and PSNR. To enable better use of 4D-sCT for guidance and dose calculation in lung-cancer radiation therapy, the CT values of 4D-CBCT and 4D-sCT were measured against the 4D-CT using mean error (ME) and mean absolute error (MAE). To ensure an accurate evaluation of the training results, the precision of the registration was measured by calculating mutual information (MI). Lastly, paired t-tests were performed in Statistical Product and Service Solutions (SPSS) software to assess significant differences between all 4D-sCT and 4D-CBCT results. Given the conduct of multiple hypothesis tests, all p-values were assessed following Bonferroni correction. When the p-value is less than 0.003, the results are significantly different. The corresponding expressions are shown below:

7 $$SSIM(X,Y) = \frac{{\left( {2{\mu}_{X} {\mu}_{Y} + C_{1} } \right)(2{\sigma}_{X} {\sigma}_{Y} + C_{2} )}}{{({\mu}_{X}^{2} + {\mu}_{Y}^{2} + C_{1} )\left( {{\sigma}_{X}^{2} + {\sigma}_{Y}^{2} + C_{2} } \right)}},$$

8 $$PSNR = 10\log_{10} \frac{{\max \left| {X\left( {i,j} \right)} \right|^{2} }}{MSE},$$

9 $$MSE = \frac{1}{M \times N}\sum\limits_{i = 1}^{M} {\sum\limits_{j = 1}^{N} {(X(i,j) - Y(i,j))^{2} } } ,$$

10 $$\begin{gathered} ME(X,Y) = \frac{1}{M \times N}\sum\limits_{i = 1}^{M} {\sum\limits_{j = 1}^{N} {(X(i,j) - Y(i,j))} } , \hfill \\ \hfill \\ \end{gathered}$$

11 $$MAE(X,Y) = \frac{1}{M \times N}\sum\limits_{i = 1}^{M} {\sum\limits_{j = 1}^{N} {|X(i,j) - Y(i,j)|} } ,$$

12 $$p_{i} = h_{i} /\left( {\sum\limits_{i = 1}^{N - 1} {h_{i} } } \right),$$

13 $$H\left( Y \right) = - \sum\limits_{i = 0}^{N - 1} {p_{i} } \log p_{i} ,$$

14 $$H\left( {X,Y} \right) = - \sum\limits_{x,y} {p_{xy} \left( {x,y} \right)\log p_{xy} (x,y)} ,$$

15 $$MI(X,Y) = H(X) + H(Y) - H(X,Y),$$

In the expression of SSIM, *X* represents 4D-CBCT and 4D-sCT, and *Y* represents 4D-CT. $\mu_{{\text{x}}}$ and $\mu_{{\text{y}}}$ denote the average pixel values of images *X* and *Y*, respectively. $\sigma_{{\text{x}}}$ and $\sigma_{{\text{y}}}$ represent the variances, whereas *C* is a regularization constant with *C*_*1*_ and *C*_*2*_ taken as (0.01 × 2000)^2^ and (0.03 × 2000)^2^, respectively. The dynamic range of the image pixels was 4095. In the expressions of mean-square error (MSE), ME, and MAE, *X* represents 4D-CBCT and 4D-sCT, whereas *Y* represents 4D-CT. *M* and *N* represent the width and height of the input images, respectively. The expression for PSNR was obtained by dividing the maximum value by the MSE. In formulas (12) (13) (14) (15), *X* and *Y* denote two images, where $h_{i}$ represents the sum of pixel points in image *Y* with gray i, *N* represents the gray level in image *Y*, and $P_{i}$ represents the probability of gray i. H(Y) denotes the entropy of an image, H(X,Y) denotes the joint entropy of *X* and *Y*. MI reflects the degree of information contained between two images, with value ranging from 0 to positive infinity. The higher the similarity or overlap between images, the smaller the joint entropy and the greater the MI. After conducting paired t-tests, statistical significance was observed in the SSIM, PSNR, ME, MAE and MI of the 4D-sCT images.

To measure the local information of CT values, the 4D-CBCT, 4D-CT, and 4D-sCT images of five patients were outlined with 35 × 35, 15 × 15, and 25 × 25 regions of interests (ROIs) in the lungs, bones, and soft tissues. The mean CT values were then measured. The CT values indicated that the mean CT value difference between 4D-sCT and 4D-CT was smaller, and the images generated based on CLCGAN had the smallest differences. Moreover, to evaluate the CT value errors of the lung tumor, the 4D-CBCT, 4D-CT, and 4D-sCT images of five patients were outlined with 15 × 15 ROIs in the region of the lung tumor. The results indicated that the CT value error of CLCGAN is smaller.

#### Dose evaluation

To assess the accuracy of dose calculations, the dose distributions of 4D-CT, 4D-CBCT, and 4D-sCT were compared and the relative percentage difference (RPD) was calculated. Each phase of 4D-CT for five tested patients was contoured for target delineation and the GTV and PTV contours averaged by ten phases were used for volumetric-modulated arc therapy planning by using a planning system (Monaco 5.1, Elekta). A prescription dose of 6000 cGy over 30 days was applied. Subsequently, the 4D-CBCT and 4D-sCT generated by both methods were rigidly registered with the reference 4D-CT, and the structure contours and treatment plans from the reference 4D-CT were copied to each image. Dose calculations were performed on all images, and dose–volume histogram (DVH) parameters were assessed for the PTV, left lung, right lung, and spinal cord. For the PTV, the dose at D98% and D2% was calculated, whereas for the spinal cord, the dose at D2% was calculated. For the left and right lungs, the lung volume was calculated at V20Gy and V5Gy, respectively.

16 $$RPD = \frac{|A - F|}{{(A + F)/2}} \times 100\% ,$$

In the expression of RPD, A represents the dose or volume of 4D-CT, and F represents the dose or volume of 4D-CBCT and 4D-sCT (Cyc, and CLC).

## Results

Tables 2 and 3 present the results of image-quality evaluation. CLCGAN improved its performance in terms of SSIM and PSNR, increasing from 0.771 and 22.31 dB to 0.980 and 29.15 dB, respectively. The ME and MAE of overall CT values also decreased from − 116.70 and 220.29 to 3.20 and 70.76. Additionally, compared with CycleGAN, CLCGAN showed an improvement of 0.11 and 0.42 dB in SSIM and PSNR, respectively, and a reduction of 0.25 and 3.39 in ME and MAE. After t tests, we found that all the improvements and reductions were statistically significant, and the improvement of CLCGAN in SSIM and PSNR also had statistical significance.

**Table 2:** Evaluation Results of Structural Similarity and Peak Signal-to-Noise Ratio

Dataset	SSIM	P value(vs 4D-CBCT)	P value(vs Cyc)	PSNR(dB)	P value(vs 4D-CBCT)	P value(vs Cyc)
4D-CBCT	0.771 ± 0.19	-	-	22.31 ± 1.21	-	-
4D-sCT(Cyc)	0.969 ± 0.05	p < 0.01	-	28.73 ± 1.57	p < 0.01	-
4D-sCT(CLC)	0.980 ± 0.02	p < 0.01	p < 0.01	29.15 ± 1.73	p < 0.01	p < 0.01

The table includes the mean ± variance of SSIM and PSNR based on 4D-CT for five patients. The significance of 4D-sCT was evaluated using paired t test, and the significance of the differences between 4D-sCT generated by the two methods was assessed

"–" indicates that no comparison was made

**Table 3:** Evaluation results of mean error and mean absolute error

Dataset	ME(HU)	P value(vs 4D-CBCT)	MAE(HU)	P value(vs 4D-CBCT)
4D-CBCT	− 116.70 ± 30.83	–	220.29 ± 337.39	-
4D-sCT(Cyc)	3.45 ± 11.31	p < 0.01	74.15 ± 71.25	p < 0.01
4D-sCT(CLC)	3.20 ± 8.25	p < 0.01	70.76 ± 68.21	p < 0.01

The table includes the mean ± variance of ME and MAE based on 4D-CT for five patients. The significance of 4D-sCT was evaluated using paired t test

"–" indicates that no comparison was made

Additionally, Table 4 illustrates the MI between the registered 4D-CBCT, the 4D-sCT generated using two methods and the 4D-CT. The results reveal that the MI between the registered 4D-CBCT and 4D-CT is only 0.735, whereas there is a substantial improvement in the accuracy of 4D-sCT (p < 0.01), with CycleGAN and CLCGAN yielding respective improvements of 0.568 and 0.588. After t tests, we found that the improvements of the 4D-sCT were statistically significant, and the improvement of CLCGAN based on CycleGAN had statistical significance.

**Table 4:** Evaluation results of mutual information

Index	4D-CBCT	4D-sCT(Cyc)	4D-sCT(CLC)
MI	0.735 ± 0.08	1.303 ± 0.08	1.323 ± 0.08
P value(vs 4D-CBCT)	–	p < 0.01	p < 0.01
P value(vs Cyc)	–	–	p < 0.01

The table includes the mean ± variance of MI based on 4D-CT for five patients. The significance of 4D-sCT was evaluated using paired t test

"-" indicates that no comparison was made

**Fig. 3:**
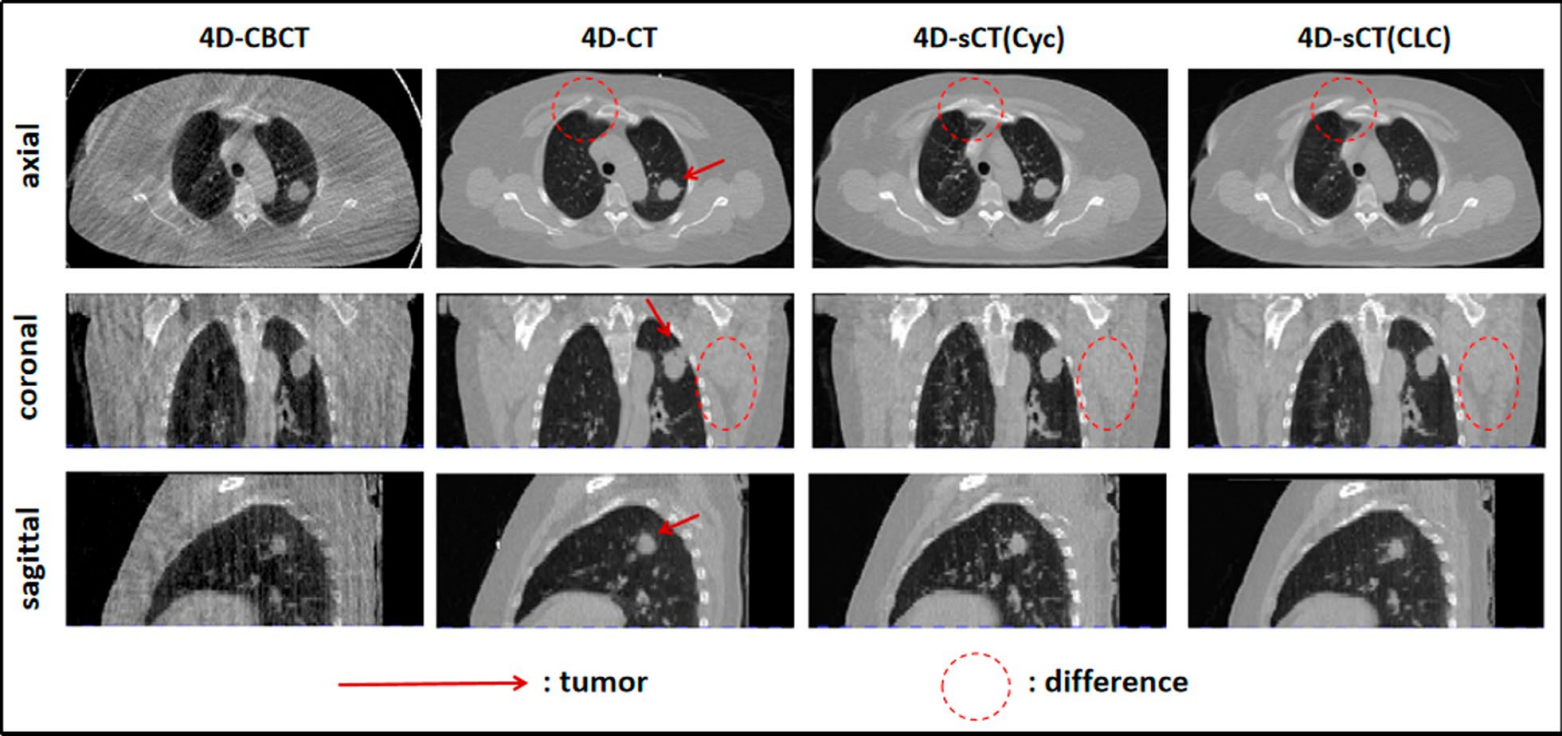
Structural Images of Patient 1 in Different Directions. The four columns represent 4D-CBCT, 4D-CT, 4D-sCT(Cyc), and 4D-sCT(CLC) images, respectively. All images are displayed at the same window width and window level

**Fig. 4:**
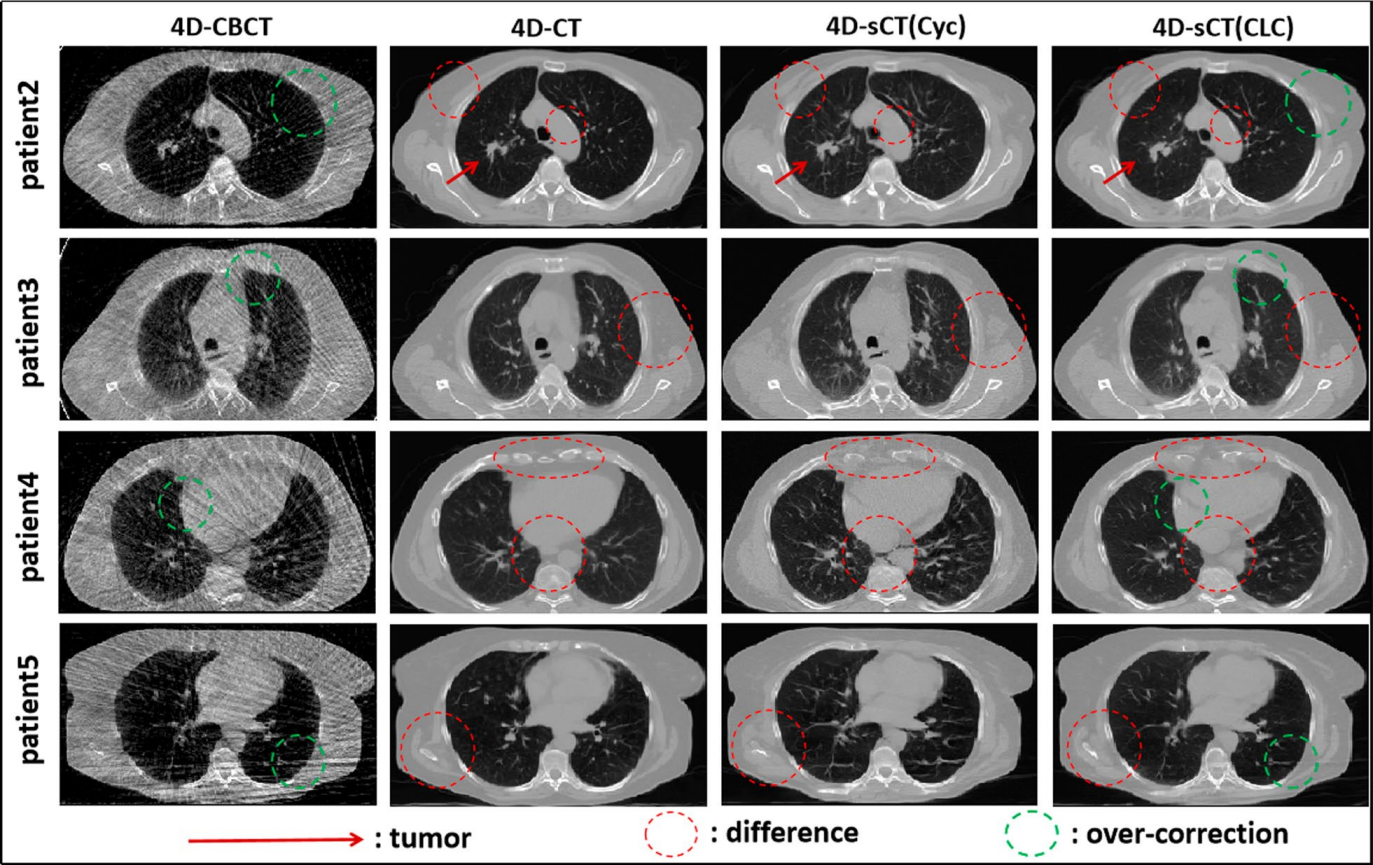
Structural images of four test patients. The four columns represent 4D-CBCT, 4D-CT,4D-sCT(Cyc), and 4D-sCT(CLC) images. All images are displayed at the same window width and window level

To illustrate the qualitative evaluation results of the images, we provided image slices of all tested patients, including 4D-CBCT, 4D-CT, and two types of 4D-sCT (Figs. 3 and 4). Figure 3 displays the slices in three directions for the first tested patient, whereas Fig. 4 shows axial slices for the remaining four patients. Under the same window and width, we observed that CLCGAN generated images with fewer artifacts in the lungs, more continuous lung texture, and clearer and more accurate details than CycleGAN. CLCGAN also performed better in restoring bone tissue and effectively recovering details of muscle and soft tissue.

**Fig. 5:**
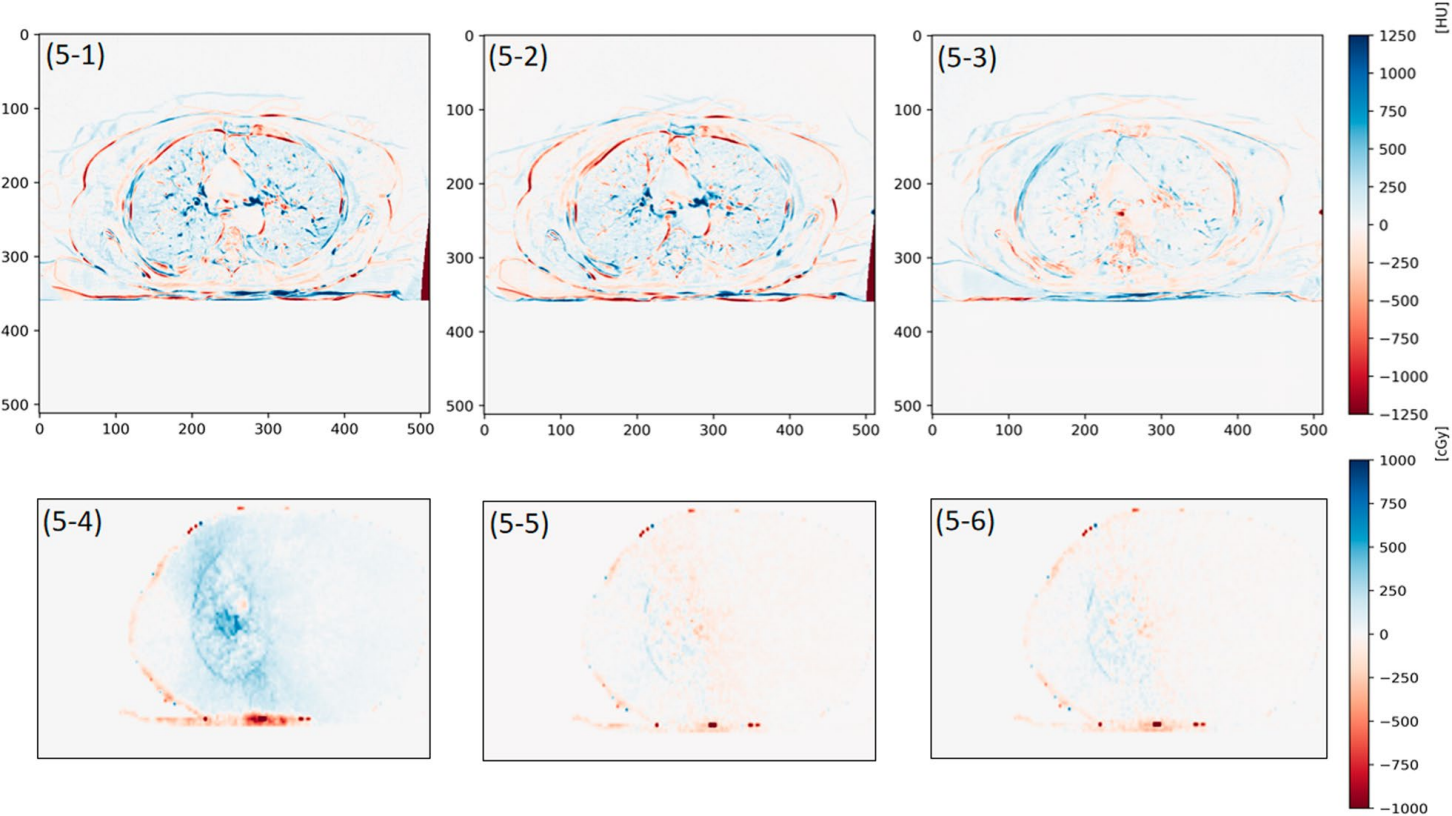
CT value difference maps and dose difference maps of Patient 2. The first row is the CT value difference, and the second row is the dose difference. **5-1** shows the difference between 4D-sCT (Cyc) and 4D-CT. **5-2** displays the difference between 4D-sCT (CLC) and 4D-CT. **5-3** represents the difference between 4D-sCT (Cyc) and 4D-sCT (CLC). **5-4** shows the difference between 4D-CBCT and 4D-CT. **5-5** displays the difference between 4D-sCT (Cyc) and 4D-CT. **5-6** displays the difference between 4D-sCT (CLC) and 4D-CT

To visually demonstrate the results of CT value correction, we selected one patient and performed subtraction between 4D-sCT and 4D-CT, as well as between the two types of 4D-sCT. Thus, we obtained axial CT value difference images (Fig. 5). Both methods were found to effectively preserve the overall structure of the 4D-sCT images. However, the CT value error was evidently smaller in the images generated by CLCGAN compared with those by 4D-CT. Particularly in the lungs and some bone structures, the difference between the images generated by CLCGAN and the 4D-CT images was smaller than that between the images generated by CycleGAN. Furthermore, we conducted a subtraction of dose distribution between the 4D-CBCT, 4D-sCT and 4D-CT for the patient, resulting in the dose difference images (Fig. 5). The findings indicate that the dose difference between the 4D-sCT generated by CLCGAN and the 4D-CT is the most minimal.

**Fig. 6:**
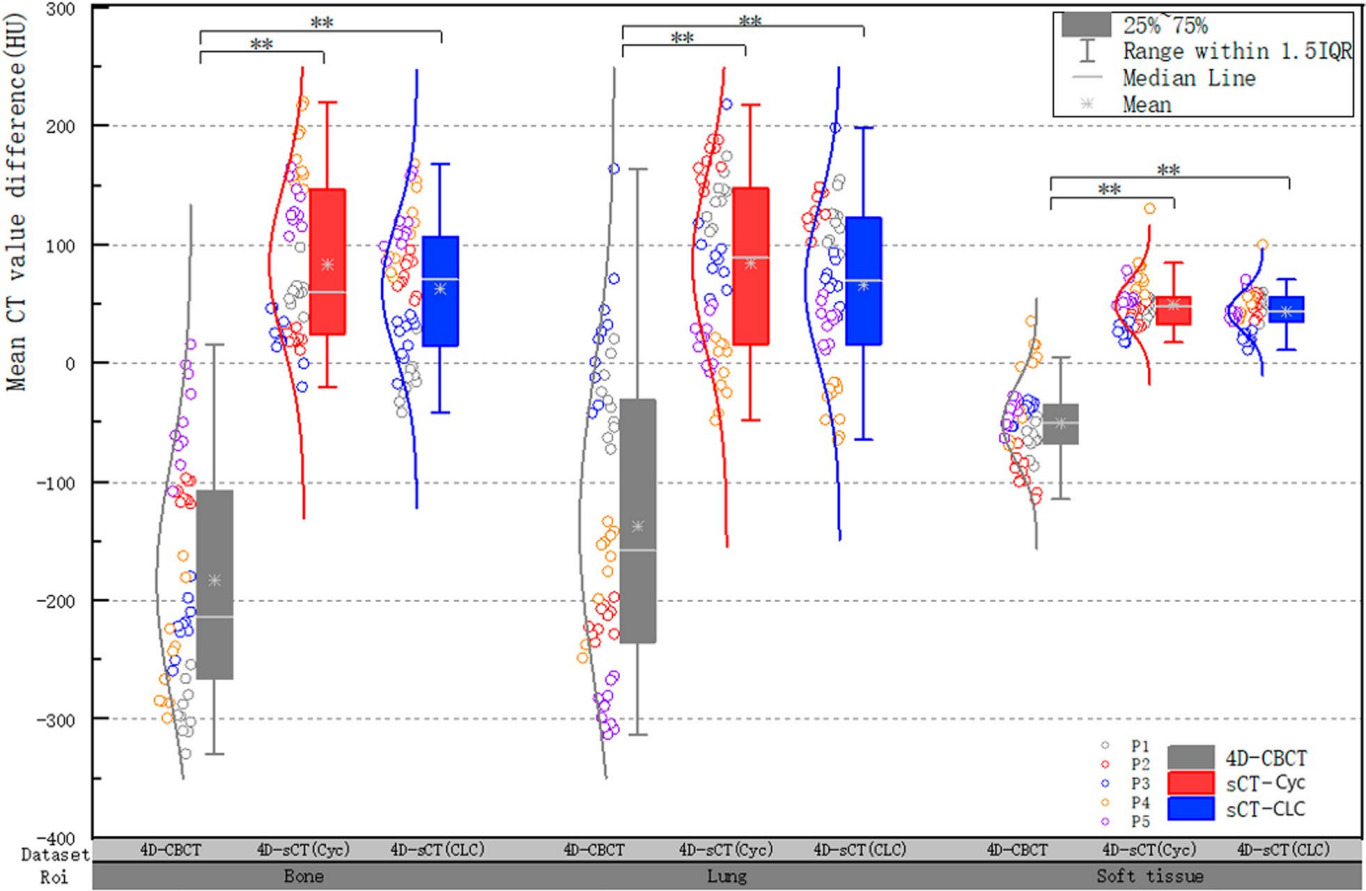
Differences in mean CT values for the regions of interests (bone, lung, and soft tissue) compared with 4D-CT

Figure 6 depicts the quantitative evaluation of the localized 3D ROI and the mean CT difference in the ROI at different phases for all tested patients under the same window and width. CLCGAN showed significant improvements in the restoration of the lung, bone, and soft tissue. The absolute mean differences from 4D-CT decreased from 137.31, 183.15, and 50.67 to 66.28, 62.91, and 43.72, respectively. Furthermore, the artifact removal of lungs, bones, and soft tissues was also significantly improved with CLCGAN relative to CycleGAN, with decreases of 18.00, 20.94, and 5.7, respectively.

The Table 5 provides the CT value errors and the errors for each patient was acquired by delineating the regions of interest for each phase. Comparing the results of 4D-CBCT and 4D-sCT with the ground truth of 4D-CT, the errors of 4D-sCT are smaller than those of 4D-CBCT, and CLCGAN demonstrates the lower errors for the lung tumor compared with CycleGAN.

**Table 5:** CT difference of lung tumor for 5 tested patients

Index	4D-CBCT	4D-sCT(Cyc)	4D-sCT(CLC)
ME	− 152.43 ± 96.85	90.24 ± 69.81	77.81 ± 76.58
MAE	153.45 ± 95.2	90.44 ± 69.54	86.71 ± 66.11

The table includes the mean errors and the mean absolute errors of CT value for the regions of lung tumor compared with 4D-CT

**Fig. 7:**
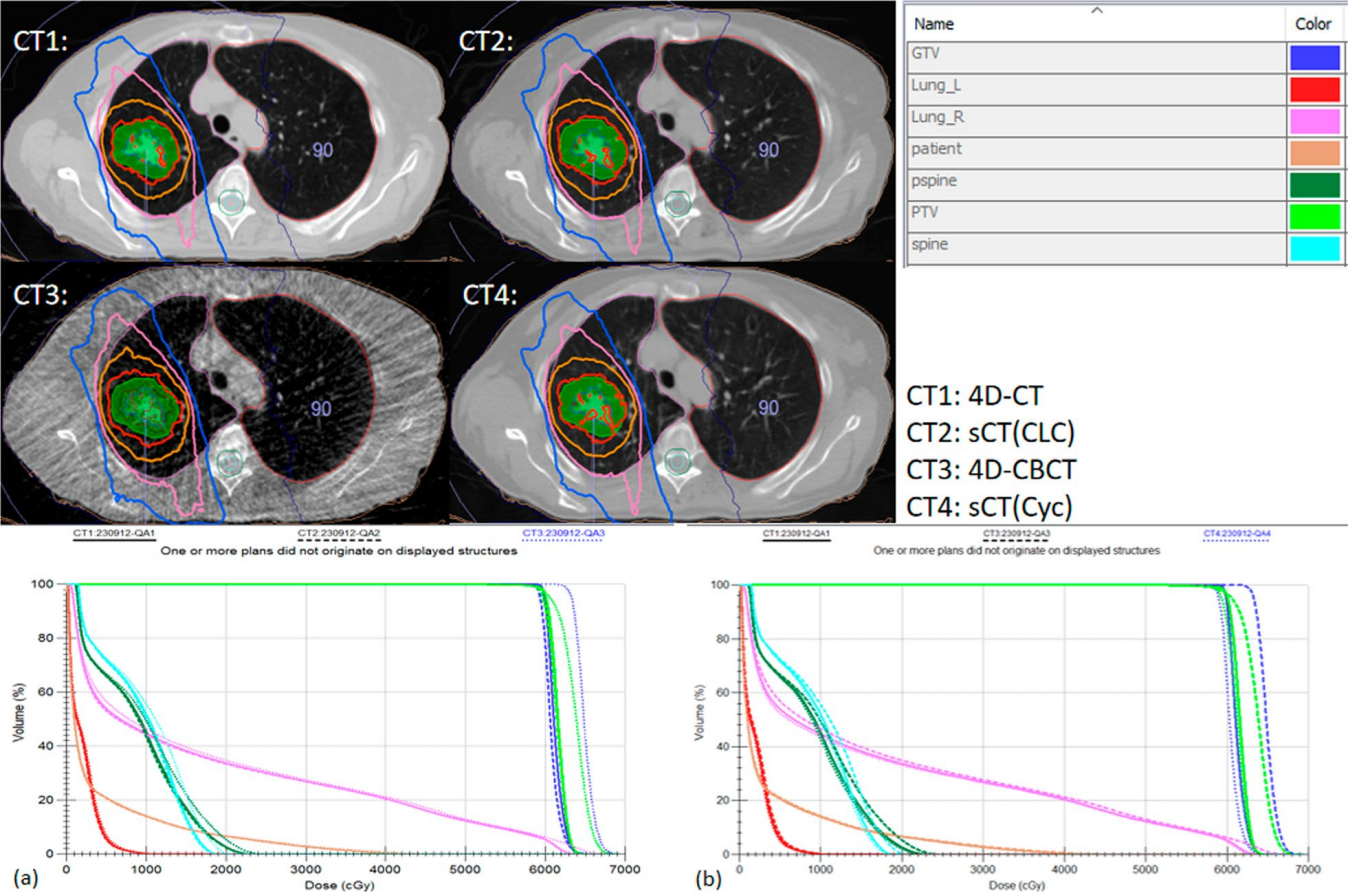
Dose distribution and dose–volume histograms (DVHs) of Patient 2: **a** 4D-CT, 4D-CBCT, and 4D-sCT (CLC); and **b** 4D-CT, 4D-CBCT, and 4D-sCT (Cyc)

The dose-calculation results are shown in Tables 6 and 7. They show the average dose difference relative to 4D-CT for the five patients and the dose difference relative to 4D-CT for each patient, respectively. In all dose-calculation results, the 4D-sCT showed a significant improvement compared with 4D-CBCT, with the relative difference close to zero. CLCGAN performed most accurately in dose calculation for the left lung (V5Gy), the right lung (V5Gy, V20Gy), the therapeutic target area (D98%), and the spinal cord (D2%). Specifically, we showed dose distribution and dose–volume histograms for one tested patient (Fig. 7). CT1, CT2, CT3, and CT4 represent the dose distribution for the reference 4D-CT, 4D-sCT (CLCGAN), 4D-CBCT, and 4D-sCT (CycleGAN), respectively. Evidently, CT2 closely resembled the dose curve of the reference CT in terms of the decrease in dose in the target region and the dose at 50% volume for the right lung and spinal cord.

**Table 6:** Average results of dose calculations for all patients

Dataset	Index	Lung_L(V5Gy)	Lung_R(V5Gy)	Lung_L(V20Gy)	Lung_R(V20Gy)	PTV(D98%)	PTV(D2%)	P_spine(D2%)
4D-CBCT	RPD(%)	9.34%	5.68%	1.08%	2.32%	1.46%	3.44%	3.66%
	vol(%)/dose(cGy)	1.334	1.054	0.268	0.344	88.4	223.4	74.62
4D-sCT(Cyc)	RPD(%)	3.58%	2.14%	0.26%	0.88%	0.76%	0.30%	0.54%
	vol(%)/dose(cGy)	0.31	0.374	0.152	0.198	44.12	18.68	11.48
4D-sCT(CLC)	RPD(%)	2.50%	1.84%	0.44%	0.86%	0.60%	0.50%	0.34%
	vol(%)/dose(cGy)	0.252	0.318	0.104	0.184	36.18	31.24	6.22

Average RPD, Volume, and dose for five patients, based on the reference 4D-CT, were calculated for 4D-CBCT, 4D-sCT (Cyc), and 4D-sCT (CLC)

**Table 7:** Results of dose calculations for all patients

RPD (%)	Dataset	Lung_L(V5Gy) (%)	Lung_R(V5Gy) (%)	Lung_L(V20Gy) (%)	Lung_R(V20Gy) (%)	PTV(D98%) (%)	PTV(D2%) (%)	P_spine(D2%) (%)
Patient01	4D-CBCT	1.0	11.5	1.4	0.0	2.9	3.5	3.9
	4D-sCT(Cyc)	0.6	5.1	0.0	0.0	0.3	0.0	0.2
	4D-sCT(CLC)	0.1	3.2	0.3	0.0	0.4	0.5	0.2
Patient02	4D-CBCT	22.2	5.4	0.0	0.7	0.7	5.2	6.6
	4D-sCT(Cyc)	13.5	2.7	0.0	1.8	1.4	0.3	2.1
	4D-sCT(CLC)	6.8	1.6	0.0	0.9	0.8	0.2	0.8
Patient03	4D-CBCT	0.3	0.4	0.7	0.4	0.1	0.8	1.0
	4D-sCT(Cyc)	1.7	0.1	0.7	0.5	0.9	0.1	0.0
	4D-sCT(CLC)	2.5	0.3	1.5	0.6	1.1	0.1	0.0
Patient04	4D-CBCT	18.0	2.0	0.0	4.2	1.9	4.2	0.5
	4D-sCT(Cyc)	2.0	0.1	0.0	0.4	0.8	0.9	0.0
	4D-sCT(CLC)	2.8	0.4	0.0	1.1	0.1	1.6	0.0
Patient05	4D-CBCT	5.2	9.1	3.3	6.3	1.7	3.5	6.3
	4D-sCT(Cyc)	0.1	2.7	0.6	1.7	0.4	0.2	0.8
	4D-sCT(CLC)	0.3	3.7	0.4	1.7	0.6	0.1	0.7

RPD for five patients, based on the reference 4D-CT, were calculated for 4D-CBCT, sCT (Cyc), and sCT (CLC)

## Discussion

4D-CBCT is an imaging technique that can display real-time lung motion. It has great practical significance in conventional and SBRT for lung cancer. However, factors such as streak artifacts caused by insufficient projection acquisition at each phase and scatter artifacts during acquisition can affect the accuracy of CT values. Such distortion can reduce the imaging quality of 4D-CBCT, make dose calculations imprecise (Fig. 7), and hinder the progress of 4D-CBCT image-based ART [24]. Therefore, we proposed a network framework called CLCGAN to utilize the feature-extraction capability of CL and thus improve the image quality of the generative model.

To reduce the problem of slight anatomical displacement caused by patient respiratory motion [34], we performed deformable registration of 4D-CT and 4D-CBCT before training. The registered 4D-CT was used as ground truth for validation. During training, we selected 10 phases. CycleGAN achieved better results in bone recovery in 4D-sCT than training with a single phase [24]. However, according to Figs. 3 and 4. severe artifacts remained in the lungs, and the lung texture was unclear. These blurry artifacts can interfere with the clinical assessment of small structures, such as blood vessels and airways. Our network learned to remove streak artifacts through feature selection before generating the images. As a result, the 4D-sCT obtained by CLCGAN can greatly reduce the streak artifacts in the lungs, the lung texture was clearer, the bone tissues were more accurate, and the results obtained were closer to the real 4D-CT. Furthermore, the quantitative evaluation listed in Table 2 shows an improvement in SSIM and PSNR for our results, which was statistically significant (P < 0.01). Due to the problems of mode collapse and unstable loss, generative adversarial networks can generate unreal, blurred, and under-diverse images [35]. CycleGAN failed to correctly recover soft tissues within some parts of the chest wall (patients 2, 3, 4, and 5) and certain high CT value regions near the spine (patient 5). Conversely, our method reduced these distortion effects (red lines in Fig. 4). However, our method is slightly over-corrected (green lines in Fig. 4), such as the brightening of the pericardial region of patient 4 caused by streak artifact is synthesized to appear even brighter. And the over-correction may be attributed to overlearning the training dataset and model’s complexity. In the future, the matter may be avoided by reducing the model’s complexity by fine-tuning the model parameters of the training dataset [36]. Moreover, Table 4 shows that the accuracy of mutual information between the registered 4D-CBCT and 4D-CT is 0.735 ± 0.08, while the two types of 4D-sCT based on CycleGAN and CLCGAN are 1.303 ± 0.08 and 1.323 ± 0.08, respectively. According to the results, the generated 4D-sCT recovers lung textures, bone, and soft tissue, leading to higher mutual information. Compared to CycleGAN, CLCGAN exhibits fewer residual artifacts and higher capability of detail recovery, thus possessing higher mutual information.

Given that 4D-CBCT can be used for ART and accurate dose calculation is needed when applying 4D-CBCT for ART, restoring the CT values while improving the image quality was necessary [9, 24]. Therefore, the ME and MAE of the overall CT value were calculated, and CT and dose difference maps were produced for the generated images, as shown in Table 3 and Fig. 5. The ME and MAE of the CT values were significantly reduced, and the dose difference was significantly decreased. The quantitative evaluation results of CT values for the local ROI are shown in Fig. 6. CLCGAN significantly improved the restoration of the lungs, bones, and soft tissues, with the smallest differences compared with 4D-CT. The improvements were more significant in the lungs and bones, consistent with the previous results from generating 4D-sCT [23, 24, 34]. Additionally, Table 5 focuses on the CT value errors of the tumor region, and the results show that the use of CLCGAN minimizes both the ME and MAE of CT value in the region of lung tumor.

4D images used for dose calculations, guiding conventional and SBRT adaptive radiation therapy have been showed to improve target repeatability while reducing target volume and radiation dose to normal tissues [9, 11]. Sonke et al. experimented on sixty-five lung cancer patients who treated with SBRT without a body frame to 54 Gy in three fractions. Even with considerable breathing motion, the PTV margins can safely be kept small [37]. Similarly, Bellec et al. demonstrated a reduction in PTV in thirty-two lung cancer patients who received a prescribed dose of 48–54 Gy in three to six fractions under the guidance of 4D-CBCT [38]. Additionally, Harsolia A et al. conducted 3D-CBCT and 4D-CBCT-guided ART to eight lung cancer patients who received a prescription dose of 63 Gy in thirty-five fractions, and 4D-CBCT achieved the best results in decreasing PTV volume and normal tissue doses [9]. In our study, a prescription dose of 60 Gy in thirty fractions was applied. The results of Table 6 and 7 were obtained by performing dose calculations on the test patients. The mean relative difference of the five patients showed a reduction in therapeutic target area and all critical organs, with all relative difference close to 0. Compared with 4D-sCT based on CycleGAN, CLCGAN further optimized the dose calculation for the V5Gy of left and right lung, V20Gy of right lung, D98% of therapeutic target area, and D2% of the spinal cord. However, it did not achieve better results for the V20Gy of left lung and D2% of therapeutic target area.

Although CLCGAN effectively corrected 4D-CBCT, it did not improve the recovery of structures such as blood vessels inside the heart, which can cause the low PSNR. Based on Table 4, the accuracy of registration is not particularly high, which may be of the reason for blood vessels recovery as well as low PSNR [39]. When removing streak artifacts, we did not consider the small amount of subtle lung texture in the feature maps [25, 26]. Moreover, faster CBCT scanners may be used in clinical practice, resulting in a shorter respiratory cycle and a larger interval between projections, which can lead to lower image quality. We did not perform experiments under such conditions, and the robustness of our method still needs to be considered. This was a limitation of our approach. In the future, it may be beneficial to collect clinical data from multiple centers or simulate datasets with sparse projections from different angles to address these limitations.

## Conclusion

We demonstrated the ability of CLCGAN to generate 4D-sCT from undersampled 4D-CBCT. Satisfactory results were obtained by quality assessment, CT value evaluation and dose calculation, with reference to 4D-CT images acquired on the same day. Therefore, the corrected 4D-CBCT based on CLCGAN can be used for dose calculation in lung cancer.

## Data Availability

The datasets used during the current study are available from the corresponding author on reasonable request.

## Abbreviations

4D-CBCT: Four-dimensional cone beam computed tomography
CLCGAN: Contrastive learning-based cycle generative adversarial networks
ART: Adaptive radiotherapy
4D-sCT: Four-dimensional synthetic computed tomography
4D-CT: Four-dimensional computed tomography
SSIM: Structural similarity index measure
PSNR: Peak signal-to-noise ratio
dB: Decibel
CycleGAN: Cycle consistent generative adversarial networks
PTV: Planning target volume
SBRT: Stereotactic body radiotherapy
DVF: Deformation vector field
CL: Contrastive learning
RUL: Right upper lobe
RLL: Right lower lobe
LUL: Left upper lobe
LLL: Left lower lobe
t-SNE: T-distributed Stochastic Neighbor Embedding
ME: Mean error
MAE: Mean absolute error
MSE: Mean-square error
MI: Mutual information
ROI: Regions of interest
DVH: Dose–volume histogram
RPD: Relative percentage difference
